# Accuracy of a Self-Administered Online Cognitive Assessment in Detecting Amnestic Mild Cognitive Impairment

**DOI:** 10.1093/geronb/gbab097

**Published:** 2021-08-01

**Authors:** Theone S E Paterson, Brintha Sivajohan, Sandra Gardner, Malcolm A Binns, Kathryn A Stokes, Morris Freedman, Brian Levine, Angela K Troyer

**Affiliations:** 1 Baycrest Health Sciences Centre, Toronto, Ontario, Canada; 2 Department of Psychology, University of Victoria, British Columbia, Canada; 3 Dalla Lana School of Public Health, University of Toronto, Ontario, Canada; 4 Rotman Research Institute, Toronto, Ontario, Canada; 5 Department of Psychology, University of Toronto, Ontario, Canada

**Keywords:** Cognitive screening, Diagnostic accuracy, eHealth, Logistic regression, Validity

## Abstract

**Objectives:**

Our aim was to validate the online Brain Health Assessment (BHA) for detection of amnestic mild cognitive impairment (aMCI) compared to gold-standard neuropsychological assessment. We compared the diagnostic accuracy of the BHA to the Montreal Cognitive Assessment (MoCA).

**Methods:**

Using a cross-sectional design, community-dwelling older adults completed a neuropsychological assessment, were diagnosed as normal cognition (NC) or aMCI, and completed the BHA and MoCA. Both logistic regression (LR) and penalized logistic regression (PLR) analyses determined BHA and demographic variables predicting aMCI; MoCA variables were similarly modeled. Diagnostic accuracy was compared using area under the receiver operating characteristic curve (ROC AUC) analyses.

**Results:**

Ninety-one participants met inclusion criteria (51 aMCI, 40 NC). PLR modeling for the BHA indicated Face–Name Association, Spatial Working Memory, and age-predicted aMCI (ROC AUC = 0.76; 95% confidence interval [CI]: 0.66–0.86). Optimal cut-points resulted in 21% classified as aMCI (positive), 23% negative, and 56% inconclusive. For the MoCA, digits, abstraction, delayed recall, orientation, and age predicted aMCI (ROC AUC = 0.71; 95% CI: 0.61–0.82). Optimal cut-points resulted in 22% classified positive, 8% negative, and 70% inconclusive (LR results presented within). The BHA model classified fewer participants into the inconclusive category and more as negative for aMCI, compared to the MoCA model (Stuart–Maxwell *p* = .004).

**Discussion:**

The self-administered BHA provides similar detection of aMCI as a clinician-administered screener (MoCA), with fewer participants classified inconclusively. The BHA has the potential to save practitioners time and decrease unnecessary referrals for a comprehensive assessment to determine the presence of aMCI.

Early detection of Alzheimer’s disease (AD) is important in the facilitation of timely clinical intervention and in moving toward the development of efficacious treatments. The prodromal stage of AD has been identified as an ideal time point for the implementation of potentially disease-modifying interventions (Freitas et al., 2013). Development and refinement of innovative tools to more accurately identify prodromal AD is an important step toward facilitating intervention and furthering research to identify disease-modifying treatments.

Amnestic mild cognitive impairment (aMCI) is defined as a recent onset memory impairment (with or without impairment in other cognitive domains) not of sufficient severity to interfere with instrumental activities of daily living (Albert et al., 2011). aMCI is representative of the cognitive phenotype of prodromal AD (Chertkow et al., 2013; Dubois & Albert, 2004). Tools currently most used in assessment for aMCI include the Montreal Cognitive Assessment (MoCA; Nasreddine et al., 2005) and Mini-Mental State Examination (Folstein et al., 1975). Both of these brief screening measures require administration and interpretation by trained health care professionals and provide useful cognitive data to clinicians and researchers. Measures that can be self-administered yet similarly inform a clinician’s in-person assessments may mitigate cost and time burdens associated with in-office cognitive screening.

Currently, there are few cognitive assessments readily available to the public for self-administration. The Self-Administered Gerocognitive Exam (SAGE; Scharre et al., 2010) can be self-administered at home, but requires scoring and interpretation by a health care professional. Three other measures offer online self-administration with algorithmic scoring/interpretation. The Brain Health Assessment (BHA; Troyer et al., 2014; www.cogniciti.com), Cognitive Function Test (CFT; Trustram Eve & de Jager, 2014; www.cft.foodforthebrain.org), and eSAGE (now BrainTest; Scharre et al. 2017; www.braintest.com) were specifically designed as online, self-administered, cognitive assessment tools that provide end-users with advice for cognitive health, including whether they should seek further assessment by a health care professional. BrainTest is validated against the SAGE in adults aged 50 and older, CFT is designed for users aged 50–70, while the BHA provides statistically normed results for those aged 20–94. Of these measures validated for use in older adults, only the BHA can be used across the adult life span. The BHA is, thus, in a unique position to provide self-administered clinical screening for cognitive impairment. To increase the clinical utility of the BHA, we aimed to determine its sensitivity to early cognitive changes associated with aMCI specifically.

The goal of this study was to validate the BHA for the detection of aMCI. Specifically, in a sample of community-dwelling older adults, we aimed to determine whether the BHA can differentiate between those with normal cognition (NC) and aMCI diagnosed using a gold-standard clinical neuropsychological assessment. Our primary objectives were to (a) determine diagnostic accuracy, including sensitivity, specificity, area under the receiver operating characteristic curve (ROC AUC), and aMCI probability categories; and (b) identify a subset of BHA variables and demographic characteristics that provide the best prediction of diagnostic group. To provide a comparison with traditional paper-and-pencil, professionally administered screening methods, we also examined the diagnostic accuracy of the MoCA. In addition, to provide evidence for convergent validity of the BHA, we examined correlations between individual BHA tasks and standard clinical tasks measuring similar constructs.

## Method

### Participants

Community-dwelling adults aged 60 or older were recruited via clinical referral from the Sam and Ida Ross Memory Clinic and via direct recruitment from the Participant Database at Baycrest Health Sciences Centre, Toronto, ON, between December 2017 and December 2018. To be eligible, participants had to have adequate visual acuity, hearing, English language proficiency, and reading ability for completion of cognitive testing. Exclusion criteria included the presence of observable clinical features of neurodegenerative diseases aside from AD (i.e., aside from aMCI), history of brain tumor, clinical stroke, seizures, traumatic brain injury with loss of consciousness longer than 30 min, more than two lacunar infarcts on brain imaging, current cancer (in treatment or palliative), untreated sleep apnea, other neurological or psychiatric disorders that may affect cognitive testing (e.g., moderate to severe depressive or anxiety symptoms), substance abuse history (in the past 6 months), and inability to use a computer. We aimed to recruit 50 individuals with NC and 50 meeting criteria for aMCI (with single or multiple domain impairments).

### Study Design

The study was designed according to the Standards for Reporting Diagnostic Accuracy statement (Bossuyt et al., 2015). Using a cross-sectional design, all referrals for neuropsychological assessment from the Memory Clinic during the study period were considered for inclusion and reviewed by a study neuropsychologist (K. Stokes). Referrals with a differential diagnosis of NC versus MCI were sent to another study neuropsychologist (T. S. E. Paterson) for assessment. Once seen for clinical neuropsychological assessment, if a patient-matched inclusion/exclusion criteria, they were approached for study participation. As the time required to complete additional study measures was not extensive, those referred for clinical assessments were not compensated unless an additional visit to Baycrest was required. Individuals from the participant database were contacted based on age criteria and prescreened for eligibility via telephone interview. Those included were administered the same core neuropsychological tests as those referred clinically. Given the total time required for database participants to complete neuropsychological and additional study measures (~4–5 h), these individuals were compensated $100 for their participation. In total, 91 individuals were referred from the Memory Clinic, and 210 individuals from the participant database were contacted. Of these 301 potential participants, 124 interested individuals met inclusion criteria and chose to participate. All participants underwent an informed consent discussion and signed a consent form. The study protocol was approved by the Research Ethics Board at Baycrest (#REB# 09-02).

### Test Methods

#### Index test description

The BHA is a self-administered, online assessment of memory and executive attention that is freely available to the public (www.cogniciti.com) and takes approximately 20–30 min to complete (Troyer et al., 2014). The BHA includes a demographic and health questionnaire and four tasks: Spatial Working Memory, where individuals match pairs of shapes across three trials; Stroop Interference, a counting variation of the original Stroop task (Stroop, 1935); Face–Name Association, an associative recognition memory task; and Letter–Number Alternation, a variation on the well-known Trail Making Test part B (TMT-B; Army Individual Test Battery, 1944). A detailed description of individual tasks and corresponding variables is provided elsewhere (Troyer et al., 2014). Participants completed the BHA individually, on a laptop with mouse, in a quiet room. They were instructed to follow on-screen instructions for the tasks and were unsupervised during administration.

The four BHA tasks are based on existing clinical and experimental tasks shown to be sensitive to subtle changes associated with age-related cognitive disorders. Implementation of these online measures was optimized for comprehension and feasibility for older adults, including practice trials to ensure proper task completion. Previous analysis of the BHA tasks found adequate internal consistency, construct validity, test–retest reliability, and alternate version reliability (Troyer et al., 2014). As administered to the public, performance on the BHA is standardized based on age-normative data. For the purposes of the current study, however, nonnormative raw data from each task were examined via statistical modeling (described below) to determine the most advantageous combination of available variables in predicting aMCI. As such, no a priori cutoff value of the BHA was imposed for the identification of individuals with aMCI.

#### Comparator test description—traditional screening measure

The MoCA is a paper-and-pencil screening measure for cognitive impairment (Nasreddine et al., 2005). This measure includes items assessing orientation, visuoconstruction, executive function, language, attention and working memory, and immediate and delayed recall. The MoCA must be administered by a trained professional and can be completed in approximately 10 min. It has a total possible score of 30 (representing the sum of cognitive domain subscores), with higher total scores indicating better performance.

#### Reference standard test description

All participants underwent a semistructured clinical interview as part of the neuropsychological assessment, and where available, collateral information was collected from a significant other concerning functional status (available for 47 of 91 participants). Neuropsychological assessments (including interview) lasted approximately 3–4 h and included standardized cognitive tasks examining intellectual ability, attention, processing speed, language, visuospatial abilities, memory, and executive function (see Supplementary Table S1 for test list; Army Individual Teat Battery, 1944; Benton & Hamsher, 1976; Delis et al, 2001; Kaplan et al., 2001; Kroenke et al., 2001; Lawton & Brody, 1969; Leach et al, 2000; Spitzer et al., 2006; Wechsler, 1987; Wechsler, 1997; Wechsler, 1999). Assessments were administered by a trained research assistant or neuropsychology practicum student. The order of administration of cognitive tests was kept generally consistent between participants. Across all participants, administration of neuropsychological tests and the BHA and MoCA was counterbalanced to control for any possible practice effects or transfer of skill between similar tasks.

The clinical neuropsychologist conducting the reference (neuropsychological) assessments (T. S. E. Paterson) did not participate in the study diagnosis. Participant data were provided to three other study neuropsychologists (K. Stokes, B. Levine, A. K. Troyer) blind to participants’ performance on the BHA and MoCA, who provided a consensus diagnosis based on the neuropsychological assessment data and history gathered during the clinical interview. Participants were diagnosed with aMCI based on previously published criteria (Albert et al., 2011), with objective memory impairment defined as deficits relative to that expected given a participant’s age, educational attainment, and intellectual status, on at least two of four memory tests. Those meeting criteria for nonamnestic MCI (Csukly et al., 2016) were excluded.

### Analyses

#### Outliers

Unprocessed BHA and MoCA data were examined for outliers. For BHA Letter–Number Alternation, two data points were determined to be anomalous. In one case, the final response time in a trial was significantly longer than expected and was replaced with that participant’s median response time. In the other case, multiple consecutive repetitions of an error were made, and the number of errors was truncated by removing subsequent error counts made on the same item.

#### Logistic models using summary scores

Logistic regression (LR) was used to estimate the accuracy of aMCI classification in two separate models: using the standardized BHA normative score (described in Troyer et al., 2014) and the total MoCA score. ROC AUCs and their 95% confidence intervals (95% CIs) were calculated using model-based predicted values. Models were fit using SAS 9.4 and SAS/STAT 14.2 Proc Logistic software (SAS Institute Inc., 2012).

#### Logistic models using variable selection

BHA and MoCA were modeled separately. Given rich data provided by the four BHA tasks (i.e., item response times, accuracy, and error rates), there were 127 BHA potential covariates for modeling the accuracy of aMCI classification, including 54 Spatial Working Memory variables, 49 Stroop variables, eight Face–Name Association variables, seven Letter–Number Alternation variables, and nine demographic and self-report variables (i.e., age, gender, education, mood, memory concerns). Because the number of potential covariates was large and there were strong correlations among many variables, a generalized linear model using penalized maximum likelihood (Friedman et al., 2010; Tibshirani et al., 2011) was used to aid variable selection. To avoid overfitting, a binomial model with a LASSO (least absolute shrinkage and selection operator) type penalty was selected (α = 1) and the elastic-net penalty λ, which controls the overall strength of the penalty, was chosen by minimizing model misclassification error (MCE) via jackknife cross-validation. All variables were standardized to mean 0 and standard deviation 1 prior to analysis. Age was forced into the model, leaving 126 other potential covariates. This penalized logistic regression (PLR) model was developed using the R package Glmnet (Hastie et al., 2021), and model coefficients were transferred to SAS software for further analysis. The regularization of the parameter estimates in the PLR model introduces some bias (shrinkage) in parameter estimates but also reduces variance estimates for predicted values.

The same methods were used to develop a MoCA model, using 12 test variables (summarizing visuospatial/executive function, attention, language, memory, and orientation) and three demographic variables (age, education, and gender). Age was forced into the model leaving 14 other potential covariates.

ROC AUC comparisons for correlated LR and PLR models were compared nonparametrically and partially for selected ranges of sensitivity.

Odds ratios (ORs) and CIs are noted for predictor variables in LR analyses. CIs are not provided for PLR analyses. As noted by Engebretsen and Bohlin (2019), statistical inference is not considered “directly possible using the elastic net, as no standard errors for the estimated parameters (i.e., slope coefficients) are computed directly.” It is also important to note that PLR may shrink ORs during the variable selection process, and as such, ORs for PLR analyses may not be directly comparable to those for LR analyses.

#### Diagnostic cut-points

A *single cut-point* was determined using model-based predicted probabilities of aMCI and varying the potential cut-point. After calculating sensitivity and specificity at each potential cut-point, the cut-point (p0) with the largest Youden’s J statistic (based on the formula sensitivity + specificity – 100%; Youden, 1950) was selected to classify each participant as low probability of aMCI (i.e., NC) or high probability of aMCI. The 95% CIs were calculated for sensitivity and specificity at p0. Because the sample has a fixed prevalence of aMCI, the selected cut-point p0 was further assessed graphically by varying the prevalence of aMCI in the population and calculating probability (“P”) of having aMCI (disorder; “D”) given the classification as aMCI (test positive; “T+”; P(D|T+)), and probability of having aMCI given classification as NC (test negative; “T−”; P(D|T−)).

*Two cut-points* were calculated using methods described in Swartz et al. (2016, *p =* .810). A sensitivity cut-point (p1) was determined by the negative predictive value (NPV, P(not D|T−), or 1 − P(D|T−)) and the negative likelihood ratio (−LR, (1 − sensitivity)/specificity). A specificity cut-point (p2) was determined by the positive predictive value (PPV, P(D|T+)) and positive likelihood ratio (+LR, sensitivity/(1 − specificity)). Three probability categories were identified by varying potential pairs of cut-points: (a) low probability of aMCI (negative index test) was defined as the model-based predicted value of aMCI less than p1; (b) high probability of aMCI (positive index test) was defined as predicted value of aMCI greater than or equal to p2; and (c) indeterminate probability of aMCI (inconclusive index test) with predicted values between p1 and p2. The proportion of the sample in each category was calculated for each model. To assess the uncertainty of the proportion in each category for the chosen cut-points, 1,000 bootstrap samples were generated for each model, stratifying by aMCI status to maintain study sample proportions. Comparison of the proportion in each category across correlated models was done using the Stuart–Maxwell test (Maxwell, 1970).

#### Convergent validity

To examine the convergent validity of BHA tasks with traditional neuropsychological measures of similar constructs, we computed Pearson correlations (*r* = 0.10 represents a small, *r* = 0.30 a medium, and *r* = 0.50 a large effect; Cohen, 1992) between BHA tasks and standard cognitive tests thought a priori to measure similar constructs (Furr, 2017). Specifically, correlations were computed between (a) BHA Spatial Working Memory number of clicks and Kaplan–Baycrest Neurocognitive Assessment (KBNA; Leach et al., 2000) Spatial Location total score; (b) BHA Stroop Interference median response time and Delis–Kaplan Executive Function System (Delis et al., 2001) Color–Word Interference Trial 3 completion time; (c) BHA Face–Name Association accuracy and each of KBNA Word List Recognition score and Wechsler Adult Intelligence Scale, Third Edition (Wechsler, 1997) Digit Symbol Coding Incidental Recall Paired Recall score; and (d) BHA Letter–Number Alternation completion time and TMT-B (Army Individual Test Battery, 1944) completion time.

## Results

### Participants

**Figure 1.**
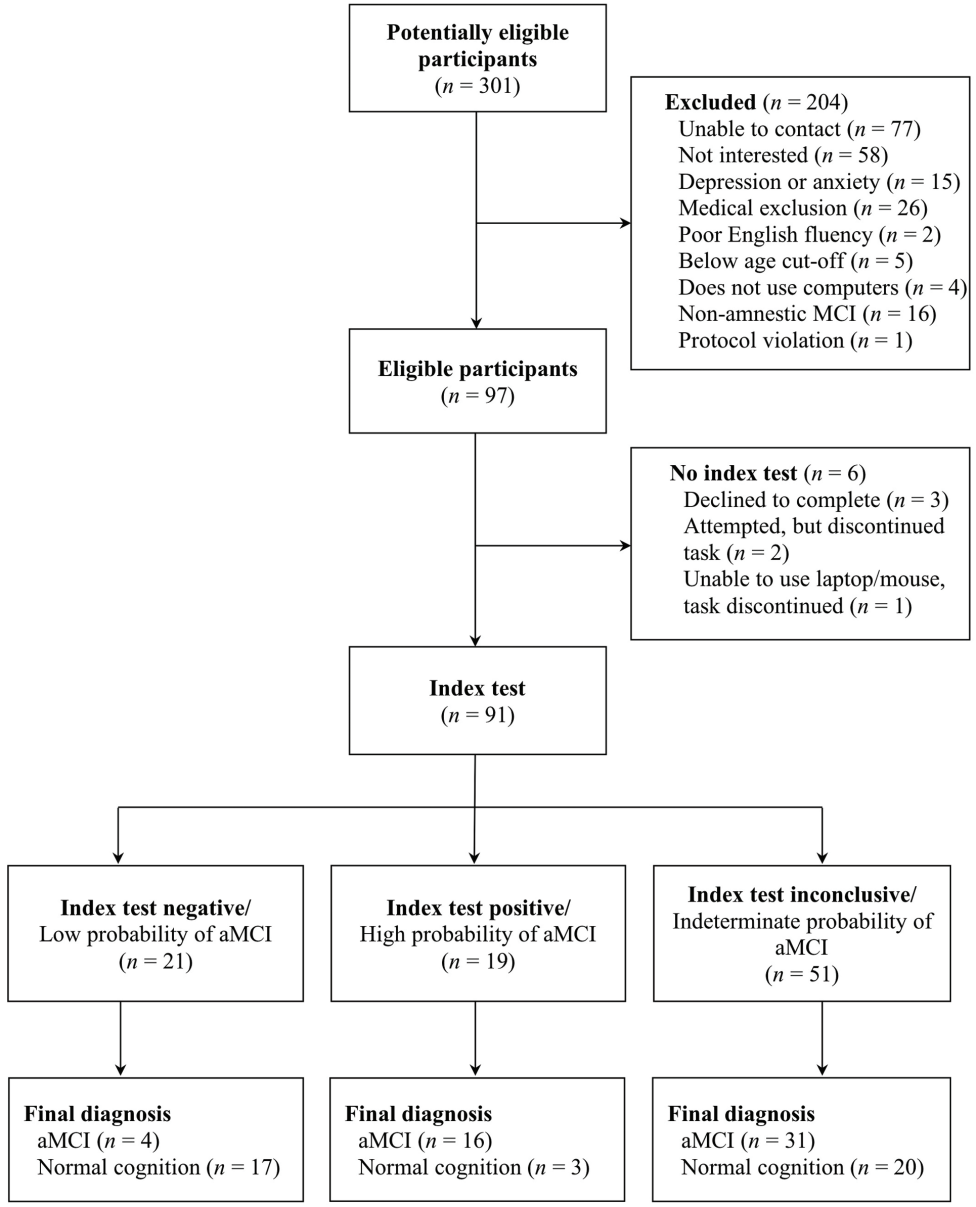
Flow diagram of participant classification by the Brain Health Assessment (index) and Neuropsychological Assessment (reference). MCI = mild cognitive impairment; aMCI = amnestic mild cognitive impairment.

One hundred and twenty-four participants were enrolled in the study. Of those, 97 were included after accounting for mood and medical exclusions. Sixteen were excluded from the sample because they met the criteria for nonamnestic MCI. Six participants also did not complete the index test and so were not included in analyses (see Figure 1 for participant flow). Data from 91 participants (51 aMCI; 40 NC) who completed all aspects of the study were included in analyses. Baseline demographic and clinical characteristics of participants in each diagnostic group are outlined in Table 1. No demographic or summary cognitive score differences were seen between memory clinic versus participant database recruits. Our sample included individuals with common comorbidities of aMCI and dementia, including hypertension, hypercholesteremia, heart disease, history of transient ischemic attack, type 2 diabetes, history of treated cancer, and self-reported anxiety, depression, attention-deficit hyperactivity disorder, and history of alcohol or substance use.

**Table 1.** Participant Demographic Information by Reference Standard Diagnostic Group

Participant characteristics	NC, *n* = 40	aMCI, *n* = 51	Effect size
Age	74 (7.0)	75 (5.7)	0.19
Gender (% female)	53	49	0.04
Education	16 (2.4)	15 (2.5)	0.12
* Range*	*10–20*	*10–20*	
ESL status (% ESL)	20	10	0.15
IQ	125 (11.4)	125 (10.2)	<0.01
* Range*	*100–145*	*92–143*	
BHA shapes *Z*-score	−0.02 (1.1)	−1.06 (1.6)	0.76
* Range*	−*2.46 to 1.69*	−*7.30 to 1.26*	
BHA faces *Z*-score	0.40 (1.0)	−0.63 (1.0)	1.03
* Range*	−*1.59 to 1.84*	−*2.20 to 1.63*	
BHA trails *Z*-score	0.06 (1.2)	−0.03 (1.0)	0.08
* Range*	−*5.68 to 1.37*	−*2.77 to 1.26*	
BHA Stroop *Z*-score	0.12 (1.2)	−0.72 (2.1)	0.49
* Range*	−*4.15 to 1.90*	−*8.72 to 1.96*	
BHA normative score	0.14 (0.7)	−0.61 (1.0)	0.85
* Range*	−*1.89 to 1.09*	−*3.59 to 0.99*	
MoCA total score	25 (2.3)	24 (2.6)	0.61
* Range*	*2–29*	*17–29*	

*Notes:* NC = normal cognition; aMCI = amnestic mild cognitive impairment; ESL = English as a second language; BHA = Brain Health Assessment; Shapes = Spatial Working Memory task; Faces = Face–Name Association task; Stroop = Stroop Interference task; Trails = Letter–Number Alternation task; MoCA = Montreal Cognitive Assessment. Unless otherwise specified, data are presented as means with standard deviations in parentheses. Effect sizes are Cohen’s *d* for *t*-tests and Cramer’s V for χ ^2^.

In relation to our primary aim, a sample size of 91 with 56% aMCI would have a 95% CI width of 24% if the sensitivity is 80% and a width of 29% if the specificity is 75% (two-sided, exact CIs; PASS, 2020). This sample size has 82% power to detect a difference of 0.17 in the ROC AUC with a null hypothesis that the AUC is 0.5 (two-sided *z*-test, α = 0.05; PASS, 2020). Additionally, for examination of individual predictors in our LR models, a sample size of 91 has 81% power to detect an OR of 2.0 or 0.5 (or detect a change of 56%–72% in the probability of aMCI) for a change in 1 standard deviation of a continuous predictor variable *X* (a small to medium effect size, two-sided, α = 0.05, *r*-squared with other predictors is 0.25, tests for the OR in LR with one normal *X* and other *X*s [Wald Test], PASS 2020).

### BHA Models

The mean normative score was 0.14 (*SD* = 0.71) for the NC group and −0.61 (*SD* = 1.01) for the aMCI group. The BHA LR model indicated that higher mean normative scores were associated with a lower probability of aMCI (*p* = .006; OR [per 1 standard deviation change in score] = 0.35; 95% CI: 0.19–0.64). The model-based ROC AUC was 0.75 (95% CI: 0.65–0.85).

**Figure 2.**
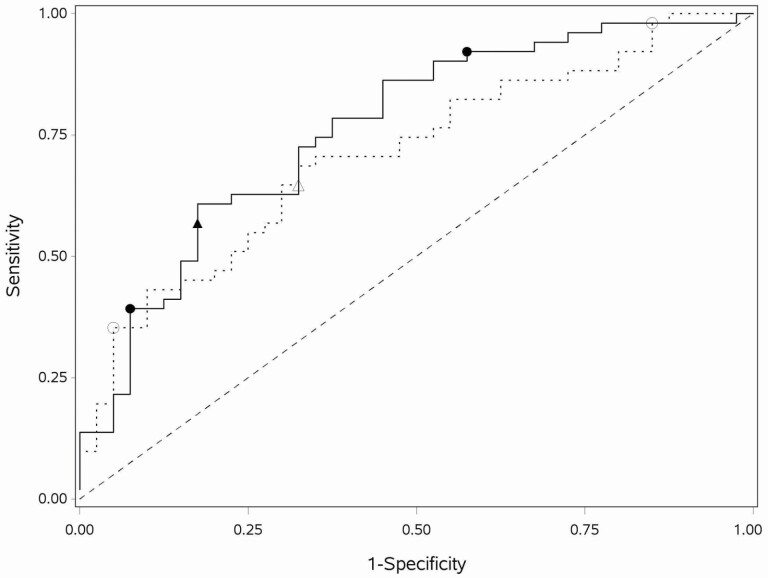
ROC curves for final BHA and MoCA PLR models. The ROC curves for the BHA (solid line) and MoCA (dashed line) PLR models with the single cut-point (solid triangle for BHA; open triangle for MoCA) and two cut-points (solid circles for BHA; open circles for MoCA). MoCA = Montreal Cognitive Assessment; BHA = Brain Health Assessment; PLR = penalized logistic regression; ROC = receiver operating characteristic.

Two variables were selected in addition to age for the BHA PLR model when MCE was minimized at λ = 0.126 (MCE = 32%). All other covariates had penalized parameter estimates equal to 0. Greater Face–Name Association task accuracy was associated with a lower probability of aMCI (OR = 0.71). More errors across all three trials of the Spatial Working Memory task (OR = 1.04) and older age (OR = 1.05) were associated with a higher probability of aMCI. The model-based ROC AUC was 0.76 (95% CI: 0.66–0.86; Figure 2). The BHA PLR model has a similar ROC AUC compared to the BHA LR model (difference = 0.01, 95% CI −0.07 to 0.09, *p* = .78).

This model classified 60% of the sample as having NC (low probability of aMCI) and 40% aMCI (high probability), given the chosen single cut-point with sensitivity of 57%, specificity of 83%, PPV of 81%, and NPV of 60%. See Table 2 for cross-tabulation of index and comparator tests by reference standard; additional details are given in Supplementary Table S2. The estimated P(D|T+) and P(D|T−) for the single cut-point solution in the sample (with a probability of aMCI of 56%), as well as for an estimated population probability of aMCI (10%; estimated from the literature; Petersen et al., 2009; Roberts & Knopman, 2013) are presented in Supplementary Table S3A (see Supplementary Figure S1 for graphical representation of estimates). Given the sample probability of aMCI is much higher than that in the population, these estimates provide information as to the expected performance of the BHA in the broader population. The selected optimal two cut-points for the BHA PLR model resulted in 23%, 56%, and 21% of the sample in the low, indeterminate, and high probability of aMCI categories, respectively. The sample prevalence and selected population prevalence classification rates and 95% CI for the two cut-point solution are presented in Supplementary Table S3B. Estimated P(D|T+) and P(D|T−) for the sample and for an estimated population probability of aMCI for the LR models are presented in Supplementary Tables S4 and S5A and B for comparison.

**Table 2.** Cross-tabulation of Index (BHA) and Comparator (MoCA) Test Results by Results on the Reference Standard (Neuropsychological Diagnosis) Based on Single Cut-Point Analyses

Test	TP	FP	FN	TN	Sensitivity (95% CI)	Specificity (95% CI)
BHA	29	7	22	33	57 (43–71)	83 (71–94)
MoCA	33	12	18	28	65 (52–78)	70 (56–84)
*Difference*					*−8*	*13*

*Notes: n* = 91 for both tests. TP = true positive; FP = false positive; FN = false negative; TN = true negative; BHA = Brain Health Assessment; MoCA = Montreal Cognitive Assessment.

### MoCA Models

The mean total MoCA score was 25.4 (*SD* = 2.3) for the NC group and 23.9 (*SD* = 2.6) for the aMCI group. The MoCA LR model indicated that higher total MoCA scores were associated with lower probability of aMCI (*p* = .007; OR = 0.52; 95% CI: 0.32–0.84). The model-based ROC AUC was 0.67 (95% CI 0.55–0.78).

Four variables were selected in addition to age for the MoCA PLR model when MCE was minimized at λ = 0.03 (MCE = 38%). All other covariates had penalized parameter estimates equal to 0. Higher scores on digits (OR = 0.84), abstraction (OR = 0.82), delayed recall (OR = 0.65), and orientation (OR = 0.87) were associated with lower predicted probability of aMCI. Older age was associated with higher predicted probability of aMCI (OR = 1.10). The model-based ROC AUC was 0.71 (95% CI 0.61–0.82; Figure 2). The MoCA PLR model had a similar ROC AUC compared to the MoCA LR model (difference = 0.04, 95% CI −0.02 to 0.11, *p* = .18).

This model classifies 50% of the sample as having NC (low probability) and 50% aMCI (high probability), given the chosen single cut-point with sensitivity of 58%, specificity of 70%, PPV of 73%, and NPV of 61% (Table 2 and Supplementary Table S2). The estimated P(D|T+) and P(D|T−) for the chosen single cut-point in the sample and for the estimated population probability of aMCI are presented in Supplementary Table S3A (and Supplementary Figure S1). The selected optimal two cut-points for the MoCA PLR model resulted in 8%, 70%, and 22% of the sample in low-, indeterminate-, and high-probability categories (see Supplementary Table 3B for estimated population probabilities).

### Statistical Comparison of BHA and MoCA

The BHA LR model has a similar ROC AUC compared to the MoCA LR model (difference = 0.08, 95% CI −0.03 to 0.19, *p* = .13). The BHA PLR model also has a similar ROC AUC compared to the MoCA PLR model (difference = 0.05, 95% CI −0.06 to 0.16, *p* = .36); in both cases, model differences are nonsignificant. The PLR ROC are the most separated in the sensitivity range 0.59–0.95 (Figure 2). The corrected partial AUC is 0.73 for the BHA PLR model and 0.66 for the MoCA PLR model, but the difference of (corrected) AUC of 0.07 is nonsignificant (*p* = .24). For the single cut-point models, Supplementary Figure S2 illustrates that the BHA PLR model has a higher P(D|T−) over a range of aMCI prevalence. After choosing two cut-points, the BHA model classifies less of the sample into the indeterminant probability category and more into the low probability category, compared to the MoCA model (Stuart–Maxwell *p* = .004).

### Convergent Validity

As given in Table 3, correlations between BHA measures and comparable traditional cognitive measures identified a priori were all significant and represented moderate to large effects, indicative of convergent validity of the BHA tasks. Scatterplots of these relationships are presented in Supplementary Figure S3.

**Table 3.** Correlations Between BHA Subtests and Traditional Cognitive Measures

BHA cognitive task	Traditional cognitive measure	Pearson *r*
Spatial Working Memory	KBNA Spatial Location	−0.38**
Stroop Interference	D-KEFS Color Word Interference	0.48**
Face–Name Association	KBNA Word List Recognition	0.56**
	WAIS-III DCS Incidental Paired	0.52**
Letter–Number Alternation	Trail Making Test Part B	0.61**

*Notes: n* = 91. BHA = Brain Health Assessment; KBNA = Kaplan–Baycrest Neurocognitive Assessment; D-KEFS = Delis–Kaplan Executive Function System; WAIS-III = Wechsler Adult Intelligence Scale Third Edition; DCS Incidental Paired = Digit Symbol Coding Incidental paired recall task.

**Correlation is significant at the 0.01 level (two-tailed).

## Discussion

There is a need for brief cognitive assessment tools that can detect early signs of cognitive decline, and online, self-administered measures provide the potential for broad reach of cognitive screening to individuals who may otherwise not receive clinical assessments. To date, few studies have attempted to validate computer and online screening tools for the detection of MCI and dementia (De Roeck et al., 2019). We examined the traditional scores calculated from the BHA and MoCA as well as models allowing selection of the most predictive variables. In both cases, the BHA and MoCA showed similar diagnostic accuracy for identifying aMCI. Modeling of BHA and demographic variables yielded an overall diagnostic accuracy of 68%, with the PLR model indicating that aMCI was predicted by age, Face–Name Association, and Spatial Working Memory variables. This makes sense, given the defining mnemonic changes of aMCI. Similar analyses of the MoCA indicated diagnostic accuracy of 67%, with the PLR model indicating aMCI was predicted by age and cognitive domains including attention, abstraction, orientation, and delayed recall. Despite the similar diagnostic accuracies, however, the BHA and MoCA differed in proportions of participants with negative, positive, and inconclusive diagnostic results, as discussed below.

Diagnosis with aMCI, of course, requires a clinical and functional evaluation (Albert et al., 2011) and would not be communicated to test-takers outside a clinical setting. However, there is an important role for screening measures to detect aMCI in clinical settings, as well as for the selection of appropriate participants for intervention trials in research settings. These varied uses of screening measures may stipulate the utility of different cutoffs for a given measure. A more inclusive cutoff with high sensitivity, and thus a low false-negative rate, is prudent in clinical settings to minimize screening-out of individuals who may benefit from a gold-standard neuropsychological assessment to better determine cognitive status and routing into available clinical intervention programs. In this case, the higher cutoff point can be used to ensure clients with aMCI are not missed. For research recruitment, on the other hand, where there is a premium on ensuring accurate sample composition, false-positive cases need to be minimized, and a cutoff emphasizing high specificity is preferable. In this case, the use of a lower cutoff will ensure only individuals most likely to have aMCI are selected into research trials.

We thus calculated optimal dual cutoff points for the BHA, maximizing both sensitivity and specificity, and allowing for determining the probability of aMCI based on test scores. Using dual cutoffs in our sample, 21% of participants were classified as high probability of aMCI (test positive), 56% as indeterminate probability (test inconclusive), and 23% as low probability (test negative). It is clear that a number of individuals diagnosed as having aMCI by neuropsychological assessment (*n* = 51) did not screen into the high probability group, and similarly, the indeterminate probability group includes some individuals not diagnosed with aMCI (*n* = 40). Interestingly, a comparison of classifications using dual cutoffs for BHA and MoCA data indicates that the BHA classifies fewer individuals inconclusively than the MoCA. The BHA appears to better differentiate between those at more moderate and lower probability for aMCI, as indicated by the Stuart–Maxwell test. The advantage seen for the BHA relative to the MoCA in the screening of higher functioning people is remarkable given that the MoCA, a clinician-administered tool, would be expected to be of better diagnostic capacity than an unsupervised test, all else being equal. Results suggest that initial screening using the BHA may save a number of individuals unnecessary additional medical visits over screening using the MoCA, which may be somewhat less precise (imprecision that may be reflected in the relatively small summary score difference seen between aMCI and NC in our sample). On the other hand, when the probability for aMCI is high, both tests yield similar results, and the MoCA may have some advantage identifying more severe cognitive impairment affecting orientation and basic processing skills, as the BHA does not measure these domains.

We also estimated classification rates based on BHA screening in the population of adults older than age 60 (in which the estimated prevalence of aMCI is ~10%; Petersen et al., 2009; Roberts & Knopman, 2013). Using our optimal dual cutoffs, the estimated rate of high-probability screens using the BHA was 10%. Similar estimation done using the MoCA indicated 8% would screen as high probability. However, in this scenario, screening using the BHA provided better differentiation of those at low and possible moderate probability than did the MoCA. Of note, population estimates are provided to show the projected accuracy of the BHA and MoCA in scenarios with a lower probability of aMCI than that seen in our sample. However, screening for aMCI should not be undertaken at a population level due to the likelihood of false positives, but rather in the context of those seeking services in a clinic setting, as they are at a higher risk of aMCI.

We also examined the convergent validity of the BHA tasks with standard neuropsychological measures of similar cognitive constructs. Results indicate good convergent validity of BHA tasks, with medium to large associations between these and their traditional neuropsychological counterparts. We have previously provided evidence for internal consistency, test–retest reliability, alternate version reliability, measurement error, and structural validity of the BHA (Troyer et al., 2014). Evidence for diagnostic accuracy and convergent validity provided in the current study adds further support for the measurement properties of the BHA. Additional research is needed to examine as-yet-untested properties, including responsiveness, discriminant validity, and cross-cultural validity.

There are some limitations to this study. We utilized a cross-sectional design that did not allow for the examination of within-subject variability in performance across time points. A longitudinal design would allow for this, but was not feasible due to the required duration and resources. We did not factor test–retest reliability in the current sample into calculations of predictive validity. However, the BHA has been found to have adequate test–retest reliability in a large sample of healthy older adults (Troyer et al., 2014). Outliers for two participants were also fit to the data, which reduced sample variability, and may have had some, likely small, impact on results. The sample size was also not large enough to have optimal power for direct comparisons between BHA and MoCA models given correlated ROC AUCs. As the main goal of this study was to determine the ability of the BHA and MoCA to differentiate aMCI from those with NC, we also excluded those with evidence of significant depression or anxiety and those with nonamnestic MCI, which may decrease the generalizability of results, as may the relatively high mean education levels seen in our sample. Given the demonstrated utility of the BHA in detecting aMCI in this study, however, future research further examining the utility of this measure in more diverse samples is certainly warranted.

## Conclusions

Accurate identification of aMCI enables early planning for patients and families and earlier implementation of interventions to potentially slow cognitive decline. The BHA is a short, self-administered, online cognitive measure with utility as a screening measure for aMCI in community-dwelling older adults. In spite of unsupervised administration of the BHA, our analyses indicate similar overall accuracy of classification as that of the MoCA, with some advantage to the BHA in identifying those with NC. Our results provide support for the validity of the BHA as a cost- and time-efficient tool that can assist in streamlining preassessment for aMCI by health care practitioners.

## Funding

This work was supported by funding from the Centre for Aging + Brain Health Innovation (CABHI) Researcher–Clinician Partnership Program (Project title: *Clinical Validation of the Cogniciti Brain Health Assessment*). Support was also received from the Saul A. Silverman Family Foundation as part of the Canada International Scientific Exchange Program (M. Freedman) and Morris Kerzner Memorial Fund (M. Freedman).

## Conflict of Interest

Malcolm A. Binns, Morris Freedman, Brian Levine, and Angela K. Troyer are members of the Cogniciti Research and Development Board, and receive consulting fees from Cogniciti. Morris Freedman is listed on a patent related to methods and kits for differential diagnosis of Alzheimer disease versus frontotemporal dementia using blood biomarkers.

## Supplementary Material

gbab097_suppl_Supplementary_Material
