# Supplementary material for: Accuracy of a Self-Administered Online Cognitive Assessment in Detecting Amnestic Mild Cognitive Impairment
Source: J Gerontol B Psychol Sci Soc Sci. 2021 Aug 1;77(2):341–50. doi: 10.1093/geronb/gbab097 (PMC8824689; doi:10.1093/geronb/gbab097)

Accuracy of a Self-Administered Online Cognitive Assessment in Detecting Amnestic Mild Cognitive Impairment

Supplementary Material

Table S1. Cognitive and Psychosocial Tasks Administered.

Test Title	Reference
Wechsler Abbreviated Scale of Intelligence	Wechsler, 1999
Vocabulary (split-half), Matrix Reasoning	
Kaplan-Baycrest Neurocognitive Assessment	Leach et al., 2000
Spatial Location, Word Lists, Complex Figure, Clocks	
Wechsler Adult Intelligence Scale, Third Edition	Wechsler, 1997
Digit Symbol-coding	
Trail Making Test Parts A and B	Army Individual Test Battery, 1944
Delis-Kaplan Executive Function System	Delis et al., 2001
Color-Word Interference Test	
Wechsler Memory Scale-Revised	Wechsler, 1987
Logical Memory I and II (single story)	
Boston Naming Test	Kaplan et al., 2001
Controlled Oral Word Association Test	Benton and Hamsher, 1976
Phonemic and Semantic Fluency	
Patient Health Questionnaire-9	Kroenke et al., 2001
Generalized Anxiety Disorder Scale-7	Spitzer et al., 2006
Lawton and Brody Activities of Daily Living Questionnaire	Lawton and Brody, 1969

Table S2. Single and Multiple Cut-Point Classifications for the Brain Health Assessment and Montreal Cognitive Assessment based on the Penalized Logistic Regression (PLR) Model.

	Diagnostic Characteristic	Single Cut Point	Two Cut Points
**Brain Health Assessment**			
	Cut point(s)*	.59	.48, .64
	Sensitivity	.57	.92
	Specificity	.83	.93
	Positive Predictive Value	.81	.84
	Negative Predictive Value	.60	.81
	Positive Likelihood Ratio	3.25	4.18
	Negative Likelihood Ratio	0.52	0.19
	Youden's J	.39	
	Misclassification Error	.32	
**Montreal Cognitive Assessment**			
	Cut point(s)*	.58	.37, .65
	Sensitivity	.65	.98
	Specificity	.70	.95
	Positive Predictive Value	.73	.90
	Negative Predictive Value	.61	.86
	Positive Likelihood Ratio	2.16	7.06
	Negative Likelihood Ratio	0.50	0.13
	Youden's J	.35	
	Misclassification Error	.33	

* Predicted probability of aMCI

Table S3A. Classification Statistics using a Single Cut-Point in Study Sample and Estimated in the Population for the Brain Health Assessment and Montreal Cognitive Assessment, based on the Penalized Logistic Regression (PLR) Model.

		**Brain Health Assessment**			**Montreal Cognitive Assessment**		
	**Probability of aMCI**	**% Classified**	**Bootstrap 95%CI**		**% Classified**	**Bootstrap 95%CI**	
**Sample**	Low	60	51.5	70.3	51	40.7	59.3
	High	40	29.7	48.4	49	40.7	59.3
**Population***	Low	79	67.2	88.2	67	53.0	78.2
	High	21	11.8	32.8	33	21.8	47.0

*Population prevalence of aMCI is estimated at 10%. CI = Confidence Interval.

Table S3B. Classification Statistics using Two Cut-Points in Study Sample and Estimated in the Population for the Brain Health Assessment and Montreal Cognitive Assessment, based on the Penalized Logistic Regression (PLR) Model.

		**Brain Health Assessment**			**Montreal Cognitive Assessment**		
	**Probability of aMCI**	**% Classified**	**Bootstrap 95%CI**		**% Classified**	**Bootstrap 95%CI**	
**Sample**	Low	23	15.4	30.8	8	2.2	13.2
	Indeterminate	56	46.2	67.0	70	61.5	80.2
	High	21	13.2	28.6	22	14.3	29.7
**Population***	Low	39	25.4	52.6	14	4.5	24.9
	Indeterminate	51	36.9	66.5	78	65.3	89.0
	High	10	2.9	18.7	8	2.9	15.4

*Population prevalence of aMCI is estimated at 10%. CI = Confidence Interval.

Table S4. Single and Multiple Cut-Point Classifications for the Brain Health Assessment and Montreal Cognitive Assessment based on the Logistic Regression (LR) Model.

	Diagnostic Characteristic	Single Cut Point	Two Cut Points
**Brain Health Assessment**			
	Cut point(s)*	.47	.35, .80
	Sensitivity	.80	.94
	Specificity	.70	.95
	Positive Predictive Value	.77	.87
	Negative Predictive Value	.74	.79
	Positive Likelihood Ratio	2.68	5.10
	Negative Likelihood Ratio	.28	.21
	Youden's J	.50	
	Misclassification Error	.24	
**Montreal Cognitive Assessment**			
	Cut point(s)*	.61	.41, .72
	Sensitivity	.47	.92
	Specificity	.80	.98
	Positive Predictive Value	.75	.88
	Negative Predictive Value	.54	.71
	Positive Likelihood Ratio	2.35	5.49
	Negative Likelihood Ratio	.66	.31
	Youden's J	.27	
	Misclassification Error	.39	

* Predicted probability of aMCI

Table S5A. Classification Statistics using a Single Cut-Point in Study Sample and Estimated in the Population for the Brain Health Assessment and Montreal Cognitive Assessment, based on the Logistic Regression (LR) Model.

		**Brain Health Assessment**			**Montreal Cognitive Assessment**		
	**Probability of aMCI**	**% Classified**	**Bootstrap 95%CI**		**% Classified**	**Bootstrap 95%CI**	
**Sample**	Low	42	24.0	50.5	65	56.0	73.6
	High	58	49.5	76.0	35	26.4	44.0
**Population***	Low	65	52.0	76.9	77	66.0	8.2
	High	35	23.1	48.0	23	12.7	34.0

*Population prevalence of aMCI is estimated at 10%. CI = Confidence Interval.

Table S5B. Classification Statistics using Two Cut-Points in Study Sample and Estimated in the Population for the Brain Health Assessment and Montreal Cognitive Assessment, based on the Logistic Regression (LR) Model.

		**Brain Health Assessment**			**Montreal Cognitive Assessment**		
	**Probability of aMCI**	**% Classified**	**Bootstrap 95%CI**		**% Classified**	**Bootstrap 95%CI**	
**Sample**	Low	15	8.8	23.1	15	8.8	23.1
	Indeterminate	68	59.3	77.5	76	67.0	84.6
	High	17	8.8	23.6	9	3.3	15.4
**Population***	Low	25	13.9	37.1	23	11.8	36.2
	Indeterminate	68	54.3	79.3	73	60.9	85.1
	High	7	2.0	14.2	4	0.8	8.7

*Population prevalence of aMCI is estimated at 10%. CI = Confidence Interval.

Table S6A. Variable List for Brain Health Assessment PLR Analysis

BHA Task	Associated Variable
Health Questionnaire	lefthanded=(handedness ='L');
Health Questionnaire	memoryconcerns2=(memory concerns='Yes');
Health Questionnaire	RelativeswithMemoryProblems2=(Relatives with Memory Problems='Yes');
Health Questionnaire	DepressionSadness2=input(substr(put(DepressionSadness,$depresson_nervous.),1,1),1.);
Health Questionnaire	NervousWorried2=input(substr(put(NervousWorried,$depresson_nervous.),1,1),1.);
Spatial Working Memory	total clicks across all three trials
Spatial Working Memory	number of clicks to complete trial 1
Spatial Working Memory	number of clicks to complete trial 2
Spatial Working Memory	number of clicks to complete trial 3
Spatial Working Memory	total RT across ALL 3 trials
Spatial Working Memory	total accuracy across ALL 3 trials
Spatial Working Memory	total clicks to complete trial across ALL 3 trials
Spatial Working Memory	total errors across ALL 3 trials
Spatial Working Memory	difference in number of clicks to complete trial 2 vs. trial 1
Spatial Working Memory	difference in number of clicks to complete trial 3 vs. trial 2
Spatial Working Memory	difference in number of clicks to complete trial 3 vs. trial 1
Spatial Working Memory	difference in number of errors in trial 2 vs. trial 1
Spatial Working Memory	difference in number of errors in trial 3 vs. trial 2
Spatial Working Memory	difference in number of errors in trial 3 vs. trial 1
Spatial Working Memory	total RT across ALL 3 trials for truncated
Spatial Working Memory	total clicks to complete trial across ALL 3 trials for truncated
Spatial Working Memory	total accuracy across ALL 3 trials for truncated
Spatial Working Memory	total errors across ALL 3 trials for truncated
Spatial Working Memory	difference in number of clicks to complete trial 2 vs. trial 1 (truncated to remove guesses before 1st correct)
Spatial Working Memory	difference in number of errors in trial 2 vs. trial 1 (truncated to remove guesses before 1st correct)
Spatial Working Memory	difference in number of clicks to complete trial 3 vs. trial 2 for truncated
Spatial Working Memory	difference in number of clicks to complete trial 3 vs. trial 1 for truncated
Spatial Working Memory	difference in number of errors in trial 3 vs. trial 2 for truncated
Spatial Working Memory	difference in number of errors in trial 3 vs. trial 1 for truncated

Spatial Working Memory	Trial 1 total RT
Spatial Working Memory	Trial 1 total clicks to complete trial
Spatial Working Memory	Trial 1 total accuracy across trial
Spatial Working Memory	Trial 1 total errors across trial
Spatial Working Memory	Trial 2 total RT
Spatial Working Memory	Trial 2 total clicks to complete trial
Spatial Working Memory	Trial 2 total accuracy across trial
Spatial Working Memory	Trial 2 total errors across trial
Spatial Working Memory	Trial 3 total RT
Spatial Working Memory	Trial 3 total clicks to complete trial
Spatial Working Memory	Trial 3 total accuracy across trial
Spatial Working Memory	Trial 3 total errors across trial
Spatial Working Memory	Trial 1 total RT truncated score (truncated to remove guesses before 1st correct)
Spatial Working Memory	Trial 1 total clicks in trial (for calculation of truncated number of clicks)
Spatial Working Memory	Trial 1 first click after truncated
Spatial Working Memory	Trial 1 total accuracy (truncated)
Spatial Working Memory	Trial 1 total clicks truncated score (truncated to remove guesses before 1st correct)
Spatial Working Memory	Trial 1 total errors truncated score (truncated to remove guesses before 1st correct)
Spatial Working Memory	Trial 2 total RT truncated score (truncated to remove guesses before 1st correct)
Spatial Working Memory	Trial 2 total clicks in trial (for calculation of truncated number of clicks)
Spatial Working Memory	Trial 2 first click after truncated
Spatial Working Memory	Trial 2 total accuracy (truncated)
Spatial Working Memory	Trial 2 total clicks truncated score (truncated to remove guesses before 1st correct)
Spatial Working Memory	Trial 2 total errors truncated score (truncated to remove guesses before 1st correct)
Spatial Working Memory	Trial 3 total RT truncated score (truncated to remove guesses before 1st correct)
Spatial Working Memory	Trial 3 total clicks in trial
Spatial Working Memory	Trial 3 first click after truncated
Spatial Working Memory	Trial 3 total accuracy (truncated)
Spatial Working Memory	Trial 3 total clicks truncated score (truncated to remove guesses before 1st correct)
Spatial Working Memory	Trial 3 total errors truncated score (truncated to remove guesses before 1st correct)
Stroop Interference	Median RT
Stroop Interference	RT total all trials

Stroop Interference	sigma (SD) RT congruent trials
Stroop Interference	sigma (SD) RT incongruent trials
Stroop Interference	sigma (SD) RT neutral trials
Stroop Interference	sigma RT for incongruent trials minus sigma RT for congruent trials
Stroop Interference	sigma RT for incongruent trials minus sigma RT for neutral trials
Stroop Interference	sigma RT for congruent trials minus sigma RT for neutral trials
Stroop Interference	median RT incongruent trials
Stroop Interference	median RT congruent trials
Stroop Interference	median RT neutral trials
Stroop Interference	median RT for incongruent trials minus median RT for congruent trials
Stroop Interference	median RT for incongruent trials minus median RT for neutral trials
Stroop Interference	median RT for congruent trials minus median RT for neutral trials
Stroop Interference	median RT incongruent trials - 1st half of trials only
Stroop Interference	median RT congruent trials - 1st half of trials only
Stroop Interference	median RT neutral trials - 1st half of trials only
Stroop Interference	RT total all trials - 1st half of trials only
Stroop Interference	median RT for incongruent trials minus median RT for congruent trials - 1st half of trials only
Stroop Interference	median RT for incongruent trials minus median RT for neutral trials - 1st half of trials only
Stroop Interference	median RT for congruent trials minus median RT for neutral trials - 1st half of trials only
Stroop Interference	median RT incongruent trials - 2nd half of trials only
Stroop Interference	median RT congruent trials - 2nd half of trials only
Stroop Interference	median RT neutral trials - 2nd half of trials only
Stroop Interference	RT total all trials - 2nd half of trials only
Stroop Interference	median RT for incongruent trials minus median RT for congruent trials - 2nd half of trials only
Stroop Interference	median RT for incongruent trials minus median RT for neutral trials - 2nd half of trials only
Stroop Interference	median RT for congruent trials minus median RT for neutral trials - 2nd half of trials only
Stroop Interference	Percent Accuracy - congruent trials
Stroop Interference	Percent Accuracy - incongruent trials
Stroop Interference	Percent Accuracy - neutral trials
Stroop Interference	Percent Accuracy - congruent trials - 1st half of trials only
Stroop Interference	Percent Accuracy - incongruent trials - 1st half of trials only
Stroop Interference	Percent Accuracy - neutral trials - 1st half of trials only

Stroop Interference	Percent Accuracy - congruent trials - 2nd half of trials only
Stroop Interference	Percent Accuracy - incongruent trials - 2nd half of trials only
Stroop Interference	Percent Accuracy - neutral trials - 2nd half of trials only
Stroop Interference	median RT congruent trials - Accurate trials only
Stroop Interference	median RT incongruent trials - Accurate trials only
Stroop Interference	median RT neutral - Accurate trials only
Stroop Interference	median RT congruent trials - 1st half of trials only - Accurate trials only
Stroop Interference	median RT incongruent trials - 1st half of trials only - Accurate trials only
Stroop Interference	median RT neutral trials - 1st half of trials only - Accurate trials only
Stroop Interference	median RT congruent trials - 2nd half of trials only - Accurate trials only
Stroop Interference	median RT incongruent trials - 2nd half of trials only - Accurate trials only
Stroop Interference	median RT neutral trials - 2nd half of trials only - Accurate trials only
Stroop Interference	Trial RT 85th percentile-congruent
Stroop Interference	Trial RT 85th percentile-incongruent
Stroop Interference	Trial RT 85th percentile-neutral
Face-Name Association	% accuracy
Face-Name Association	ratio of correct to total responses
Face-Name Association	Total Hits (correct positive response)
Face-Name Association	Total Correct rejections (correct negative response)
Face-Name Association	Total False Alarms (incorrect positive response)
Face-Name Association	Total Misses (incorrect negative response)
Face-Name Association	Total RT for test
Face-Name Association	Median RT for test
Letter-Number Alternation	total RT in seconds
Letter-Number Alternation	Total RT
Letter-Number Alternation	% accuracy (2nd part only - clicks 9 through end of test)
Letter-Number Alternation	number of clicks to complete trial (no cap, no scale, theoretic infinite max)
Letter-Number Alternation	% accuracy of clicks over whole trial (similar to number of clicks, but on a scale 0-100%)
Letter-Number Alternation	% accuracy of clicks - 1st 8 clicks only
Letter-Number Alternation	number of clicks to complete 2nd half of test (total number of clicks - 8)

Table S6**B**. Variable List for Montreal Cognitive Assessment PLR Analysis

**BHA Task**	**Associated Variable**
Visuoconstruction/Executive Function	Visual/Executive subscore (out of 5)
Naming	Naming score (out of 3)
Attention	Digits score (out of 2)
Attention	letter tapping score (out of 1)
Attention	serial 7s score (out of 3)
Attention	Attention subscore - sum of Digits, Letter Tapping, Serial 7s (out of 6)
Language	sentence repetition score (out of 2)
Language	phonemic fluency score (out of 1)
Language	Language subscore - sentence repetition, phonemic fluency (out of 3)
Abstractions	abstraction score (out of 2)
Delayed Recall	delayed recall score (out of 5)
Orientation	Orientation score (out of 6)

Figure S1. BHA and MoCA - P(D|T+) and P(D|T-) versus prevalence of aMCI in the population.

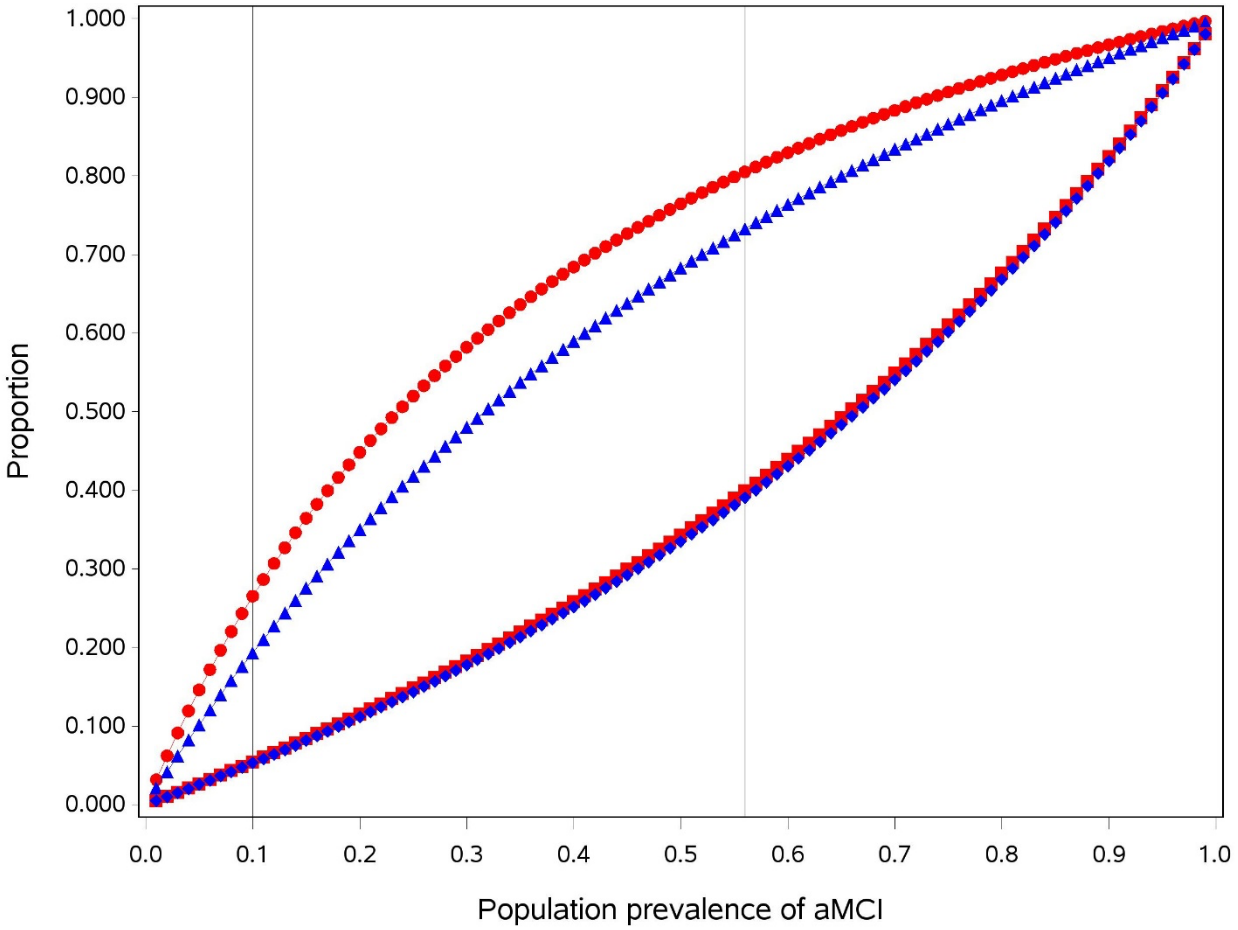

The BHA P(D|T+) (red dots) is higher than the MoCA P(D|T+) (blue triangles) for most of the range of population prevalence of aMCI. However the BHA P(D|T-) (red squares) is similar to the MoCA P(D|T-) (blue diamonds). The vertical bars at 0.10 and 0.56 are located at the estimated population prevalence and the sample prevalence, respectively. See Tables S2 for additional summaries related to these prevalence values.

Figure S2. Rule In (Sensitivity) and Rule Out (Specificity) Plots Comparing PLR Model Two Cut Point Solutions for the BHA (Red) and MoCA (Blue).

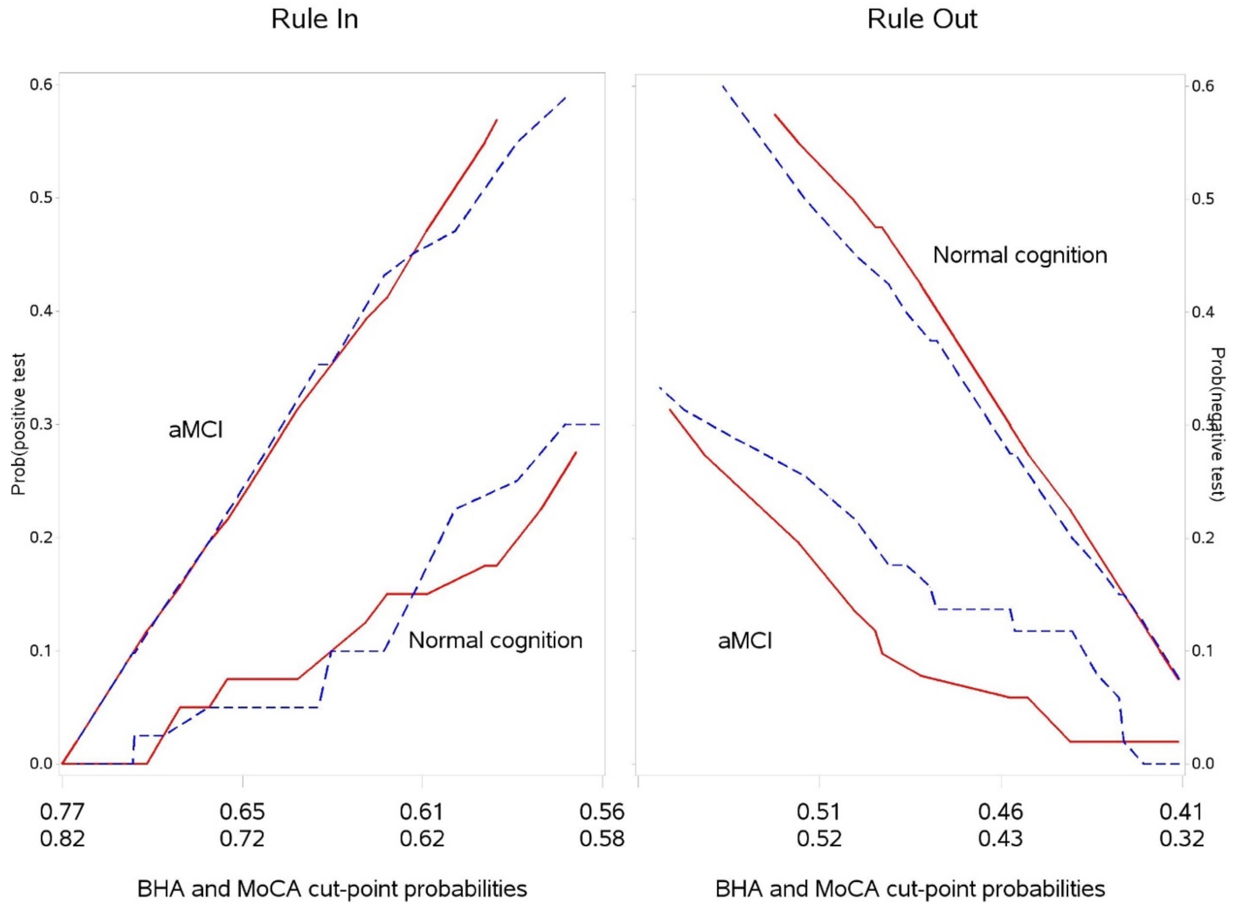

Functions of sensitivity and specificity are plotted for the BHA (red) and MoCA PLR models. The upper lines in the Rule In panel are sensitivity (ruling in the aMCI group) and the lower lines are 1-specificity (ruling in the normal cognition group). The upper lines of the Rule Out panel are specificity (ruling out the normal cognition group), and the lower lines are 1-sensitivity (ruling out the aMCI group). For both the Rule In and Rule Out panels the x-axis was calculated in polar co-ordinates (the angle in radians of arctan of sensitivity/specificity) after varying the single cut-point probabilities. The x-axis is labeled with these cut-point probabilities.

The two models are similar in ruling in the aMCI and normal cognition groups. But the BHA performs better at ruling out these groups. (See also single cut point specificities in Tables S2).

Figure S3. Scatterplots of BHA Subtests and Traditional Neuropsychological Measures of Similar Constructs.

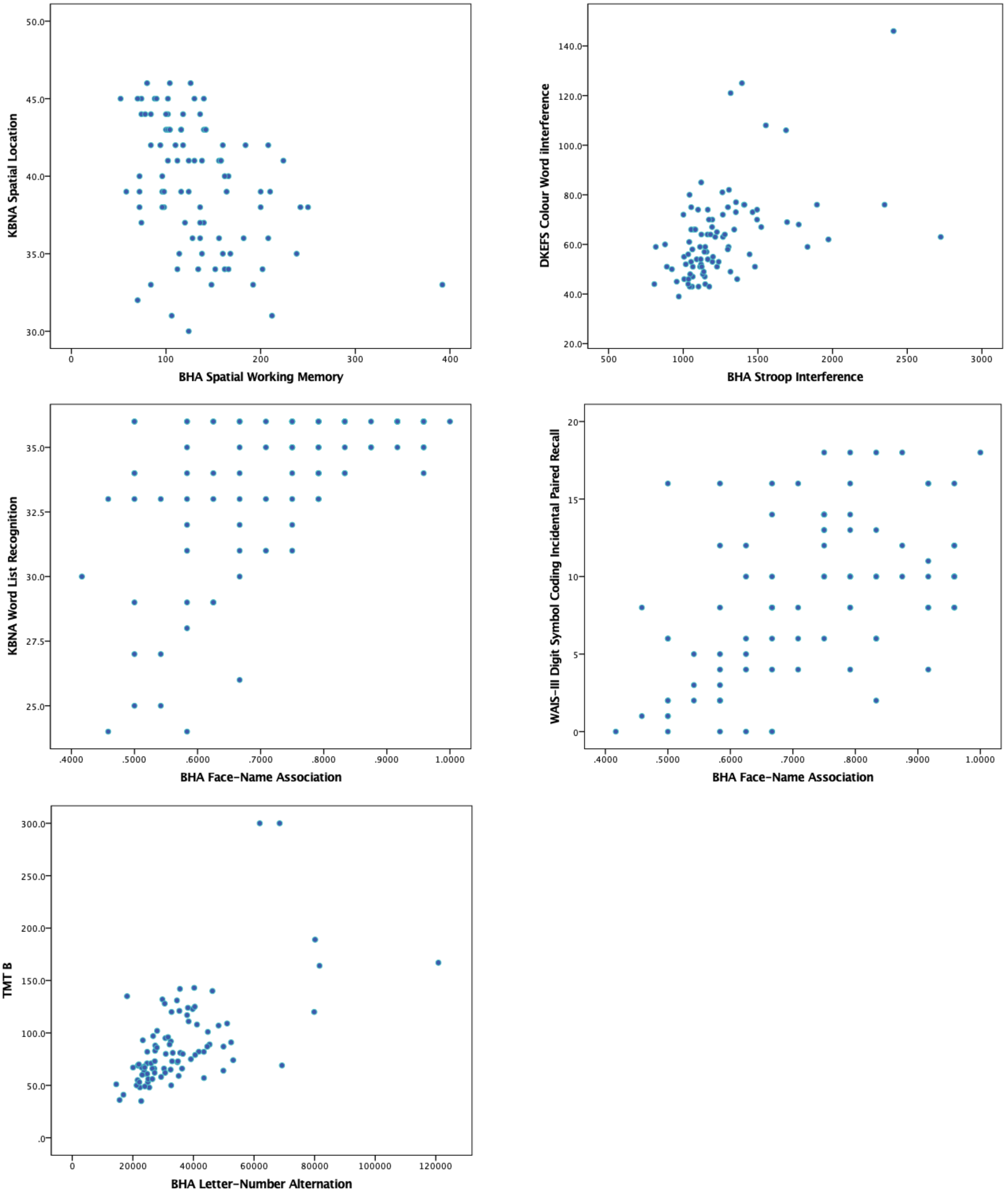

Supplement: gbab097_suppl_Supplementary_Material [file gbab097_suppl_supplementary_material.pdf]
